# Single-indicator-based Multidimensional Sensing: Detection and Identification of Heavy Metal Ions and Understanding the Foundations from Experiment to Simulation

**DOI:** 10.1038/srep25354

**Published:** 2016-05-05

**Authors:** Yumin Leng, Sihua Qian, Yuhui Wang, Cheng Lu, Xiaoxu Ji, Zhiwen Lu, Hengwei Lin

**Affiliations:** 1 Ningbo Institute of Materials Technology & Engineering (NIMTE), Chinese Academy of Sciences, Ningbo 315201, China; 2 College of Physics and Electronic Engineering, Nanyang Normal University, Nanyang 473061, China

## Abstract

Multidimensional sensing offers advantages in accuracy, diversity and capability for the simultaneous detection and discrimination of multiple analytes, however, the previous reports usually require complicated synthesis/fabrication process and/or need a variety of techniques (or instruments) to acquire signals. Therefore, to take full advantages of this concept, simple designs are highly desirable. Herein, a novel concept is conceived to construct multidimensional sensing platforms based on a single indicator that has capability of showing diverse color/fluorescence responses with the addition of different analytes. Through extracting hidden information from these responses, such as red, green and blue (RGB) alterations, a triple-channel-based multidimensional sensing platform could consequently be fabricated, and the RGB alterations are further applicable to standard statistical methods. As a proof-of-concept study, a triple-channel sensing platform is fabricated solely using dithizone with assistance of cetyltrimethylammonium bromide (CTAB) for hyperchromicity and sensitization, which demonstrates superior capabilities in detection and identification of ten common heavy metal ions at their standard concentrations of wastewater-discharge of China. Moreover, this sensing platform exhibits promising applications in semi-quantitative and even quantitative analysis individuals of these heavy metal ions with high sensitivity as well. Finally, density functional theory calculations are performed to reveal the foundations for this analysis.

Multidimensional sensing devices, which offer advantages in accuracy, diversity and capability for the simultaneous detection and discrimination of multiple analytes, have received increasing attention in recent years^1^,^2^,^3^,^4^,^5^,^6^,^7^,^8^,^9^,^10^,^11^,^12^,^13^,^14^,^15^,^16^,^17^,^18^,^19^,^20^,^21^,^22^,^23^,^24^. Traditionally, three strategies are often employed to construct multidimensional sensing systems: i) combining a variety of cross-reactively colorimetric or fluorometric indicators (i.e. sensor array approach)^1^,^2^,^3^,^4^,^5^,^6^,^7^,^8^,^9^,^10^,^11^,^12^; ii) mechanically incorporating several, such as mass-sensitive, capacitive and calorimetric transducers onto a single chip (i.e. smart chip approach)^13^,^14^,^15^; and iii) integrating fluorescent, phosphorescent, light-scattering, absorbing, and/or electrochemiluminescent (ECL) reporters on a molecule or a nanoparticle (i.e. lab-on-a-molecule/nanoparticle approach)^16^,^17^,^18^,^19^,^20^,^21^,^22^,^23^,^24^. These efforts have made great progress toward developing multidimensional sensing systems, however, they either require complicated synthesis/fabrication process and/or need a variety of techniques (or instruments) to acquire sensing signals, thus limiting their widespread applications. In order to take full advantages of the concept of multidimensional sensing, more simple design strategies are highly desirable and have in fact emerged recently. For example, Ouyang *et al*. reported a visual sensor array for recognition and analysis of proteins using two types of fluorescent gold nanoclusters^25^. Song *et al*. designed and fabricated microchips for recognition of metal ions based on a single indicator *via* multiple fluorescent channels^26^,^27^. Encouraged by these advances, we are interested in seeking even more simple but general strategies to construct multidimensional sensing systems.

Inspired by the knowledge that certain indicators show distinct color or fluorescent responses to different analytes^25^,^26^,^27^,^28^,^29^, in this current study, a triple-channel-based multidimensional sensing platform is proposed to fabricate through extracting more hidden information, such as red, green and blue (RGB) alterations, from the color or fluorescent responses of a single indicator. As a proof-of-concept study, dithizone was taken as an example of the single indicator. Through extracting RGB color alterations of dithizone with the addition of diverse metal ions, a triple-channel based multidimensional sensing platform could in principle be fabricated. Importantly, much better sensing performances are achieved with assistance by the addition of cetyltrimethylammonium bromide (CTAB), a surfactant that is known for hyperchromicity and sensitization to a probe. This sensing platform is found to be excellent in the detection and identification of ten common heavy metal ions at their standard concentrations of wastewater-discharge of China. In addition, this sensor also shows great potentials in semi-quantitative and even quantitative analysis each of these heavy metal ions with high sensitivity. The approach to multidimensional sensing systems is considered to be maximally simplified, including no need complicated synthesis, fabrication and utilization of expensive instruments.

The interactions between dithizone and heavy metal ions had solely been investigated from experimental characterization^30^ or conjecture^31^. Herein, the experimental means and density functional theory (DFT) calculations are performed to clarify the nature of interactions between dithizone and diverse metal ions. The accurate chelates of dithizone products and nine heavy metal ions are determined at B3LYP/6-31G* level. The corresponding frontier molecular orbital energies (i.e. HOMOs and LUMOs) and electronic distributions of the optimized chelates are also determined. More importantly, the calculated HOMO-LUMO gaps are found in good agreement with the experimental data, confirming the reliability of the optimized configurations. The combination of experimental characterizations and theoretical calculations demonstrate that the distinct color responses of the probe to metal ions result from the different interactions, electron distributions and transitions.

## Results

**Figure 1:**
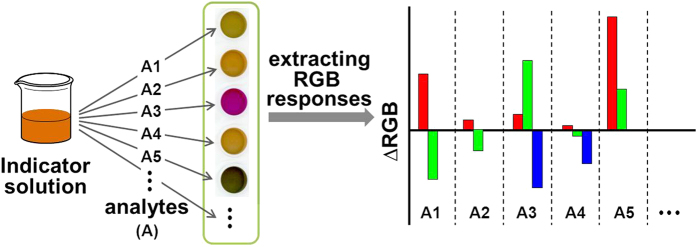
Schematic illustration of the concept of a single-indicator-based triple-channel multidimensional sensing platform.

It’s known that certain indicators show distinct color or fluorescence responses to different analytes^25^,^26^,^27^,^28^,^29^. For example, the dithizone and CTAB co-modified gold nanoparticles had been found to respond ten types of heavy metal ions with different colors in our previous study^29^. Based on this knowledge, a triple-channel-based multidimensional sensing platform could be attempted to fabricate through simply extracting red, green and blue (RGB) alterations from the indicator’s color responses with the addition of diverse analytes, as shown in Fig. 1. These RGB alterations are also applicable to standard statistical methods (e.g. Hierarchical clustering analysis (HCA) and principal component analysis (PCA)) for further evaluating the capability of such a sensing platform in detection and discrimination of multiple analytes.

To do a proof-of-concept study, dithizone was taken as an example in this study, which had been reported showing different color responses to diverse metal ions^32^,^33^. Given requirements of the practical application, ten common heavy metal ions (i.e. Hg^2+^, Cd^2+^, Pb^2+^, Cr(VI), Co^2+^, Ni^2+^, Cu^2+^, Zn^2+^, Mn^2+^, and Ag^+^) at their standard concentrations of wastewater-discharge of China (Supplementary Information (SI) Table S1) are evaluated^34^. As shown in Fig. S1 (SI), dithizone does show different color responses to six of the selected ten heavy metal ions (i.e. Co^2+^, Ni^2+^, Cu^2+^, Zn^2+^, Mn^2+^, and Ag^+^), but not to four of them (i.e. Hg^2+^, Cd^2+^, Cr(VI), and Pb^2+^), might be due to their relatively low concentrations of wastewater-discharge standard. Through extracting the RGB alterations from these color responses, a triple-channel sensing platform is fabricated, which clearly shows different recognition patterns for those six metal ions that can produce color changes to dithizone (Fig. S2 in SI). Moreover, when these RGB alterations are subjected to standard statistical analysis (e.g. HCA and PCA), similar results as those triple-channel recognition patterns are observed (Fig. S3 in SI).

Although the above results verified our concept, the relatively poor detection performance may restrict its practical applications. However, keeping in mind that the sensitivity of a probe might be significantly improved with the presence of surfactants (i.e. well-known sensitization effects of surfactants)^35^,^36^,^37^,^38^,^39^, and a cationic surfactant CTAB was attempted to sensitize dithizone’s response to metal ions. It’s astonishing to us that much stronger responses of dithizone to the ten heavy metal ions are observed with the addition of CTAB. The sensitization effects of CTAB here probably can be ascribed to its solubilization capacity to dithizone in aqueous solution, and meanwhile forming a microenvironment that favouring interaction between metal ions and the probe^35^,^36^,^37^,^38^,^39^.

To obtain better sensing performances to metal ions with the dithizone and CTAB ensemble, further optimized experiments are performed. First of all, 30 μM of dithizone was selected and fixed (for observing a clearly colored solution), and different ratios of CTAB to dithizone were examined. As seen in Fig. S4 (SI), the color responses (described by the total Euclidean distances, EDs, *i.e*. square root of the sums of the squares of the ΔRGB values) of the mixture to 5 μM Cd^2+^ are found to be increased and reach a maximum at the ratio of 3:1 (CTAB to dithizone), and then drop down at higher ratios. Secondly, pH influences on the color responses of the dithizone and CTAB mixture to metal ions were investigated. Experimental results exhibited that higher pH conditions (e.g. pH > 7) are in favour of the recognition of metal ions, but precipitations tend to appear due to the formation of metal hydroxides if pH > 8. Hence, a 90 μM CTAB and 30 μM dithizone mixture in pH 7.4 buffer is used as the optimal condition for the detection of metal ions in the following study.

**Figure 2:**
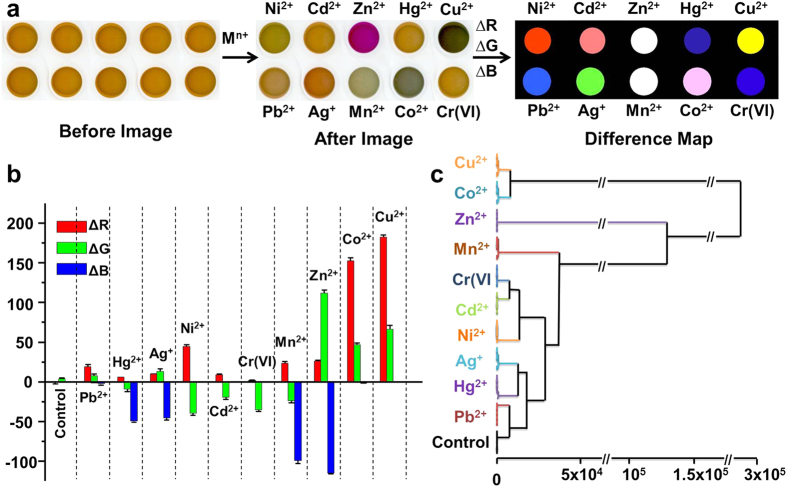
Performances of the single-indicator-based sensing platform to multiple metal ions. (**a**) Color images of the dithizone-CTAB mixture before and after exposure to ten heavy metal ions at their standard concentrations of wastewater discharge of China, and the color difference map. For purposes of visualization, the color range of the difference map was expanded from 4 to 8 bits per color (RGB range of 4–19 expanded to 0–255). (**b,c**) Recognition patterns (**b**) and HCA (**c**) for the ten heavy metal ions at their wastewater discharge standard concentrations of China and a control based on their corresponding ΔRGB values obtained from the “before” and “after” images. No confusions or errors in classification were observed in 66 trials. Error bars in (**b**) represent standard deviations of six parallel measurements.

The color responses of the single indicator (i.e. dithizone, 30 μM) in the presence of CTAB (90 μM) to the ten common heavy metal ions at their wastewater-discharge standard concentrations of China are investigated. For further reducing the instrumental requiements, an ordinary flatbed scanner (or even a digital camera) is used to acquire digital images. As shown in Fig. 2a, from the color profiles of the dithizone-CTAB mixture in the absence (named “before” images) and presence (named “after” images) of metal ions, distinctive color changes are observed to each of the ten heavy metal ions, even by naked eye. For quantitative comparisons of these color changes, a difference map can be acquired by taking the difference of the RGB values from the “before” and “after” images.

Through exctracting the RGB alterations of the dithizone and CTAB mixture after addition of metal ions (the full data set are summarized in Table S2 of SI), a triple-channel sensing platform could thus be constructed. To probe reproducibility of the sensing system and further statistical analysis, sextuple data are acquired. As shown in Fig. 2b, the ten heavy metal ions exhibit distinct ΔRGB recognition patterns (can also be called fingerprints) at their wastewater-discharge standard concentrations with good reproducibility, which demonstrates the excellent detection and discrimination capability of this sensing system. The multiple dispersion of this colorimetric sensor data requires a classification algorithm that uses the full dimensionality of the data. Herein, HCA, which is a model-free method based on the grouping of the analyte vectors according to their spatial distances in their full vector space is employed^40^,^41^. On the basis of the clustering of the sensor response data in the three-dimensional ΔRGB color space (Table S2 in SI), dendrograms formed by HCA are depicted in Fig. 2c. Remarkably, all the ten heavy metal ions and a control are accurately classified with no errors out of 66 cases. This three-dimensional response patterns are also explored by PCA, which is a statistical treatment used to reduce multidimensional data for easier interpretation^42^. The resulting two-dimensional PCA score plot (Fig. S5 in SI) shows clear clustering of the data using only the first two principal components (representing 91.5% of the total variance), with excellent discriminatory capacity. The large distance between clusters in the PCA score plot reflects significantly differential responses of the sensing system to heavy metal ions at their wastewater-discharge standard concentrations.

**Figure 3:**
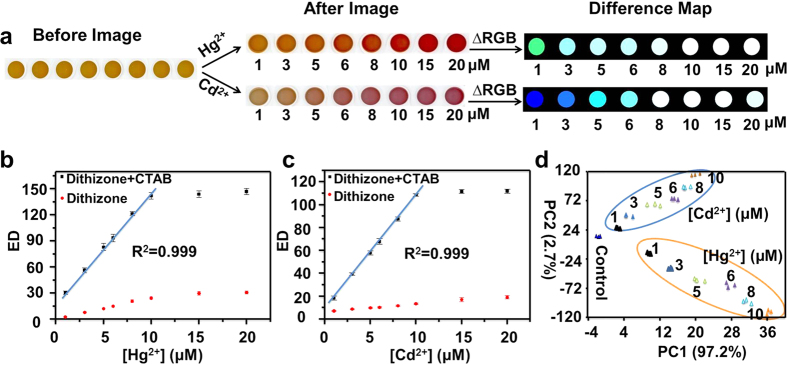
Performances of the single-indicator-based sensing platform to individuals of metal ions. (**a**) Color images of the dithizone-CTAB mixture before and after exposure to different concentrations of Cd^2+^ and Hg^2+^, along with the color difference maps. For purposes of visualization, the color difference maps were expanded from 4 to 8 bits per color (RGB range of 4–19 expanded to 0–255). (**b**,**c**) The total Euclidean distances of the dithizone solution in the absence and presence of CTAB versus different concentrations of Hg^2+^ (**b**) and Cd^2+^ (**c**). (**d**) PCA plot for the discrimination of Hg^2+^ and Cd^2+^ at different concentrations based on the RGB alterations of the dithizone-CTAB system. The experiments were performed in triplicate with 30 μM dithizone in the presence of 90 μM CTAB in 10 mM HEPES buffer at pH 7.4. The error bars in Fig. (**b**,**c)** represent the standard deviations of triplicate experiments.

To further explore capabilities of this sensing platform, such as responses at other concentrations rather than wastewater-discharge standards, potentially quantitative analysis and limit of detection (LOD), the color responses of dithizone-CTAB mixture with the addition of an individual metal ion at various concentrations are examined (Hg^2+^ and Cd^2+^ are taken as examples). As shown in Fig. 3a, more and more intense color responses are observed with increasing concentrations of the two metal ions up to about 10 μM, and these color changes can further be quantitatively indicated with total EDs (Fig. 3b,c). Importantly, nice linear fitting curves are obtained with excellent correlations (R^2^ = 0.999) between the total EDs and the concentrations of the two representative heavy metal ions from 1 to 10 μM, which actually provide possibilities for quantitative analysis. By means of extrapolating these fitting curves, the LODs are estimated to be 0.13 μM and 0.14 μM for Hg^2+^ and Cd^2+^, respectively (see Table S3 in SI for fitting parameters), both being well lower than their concentrations of wastewater-discharge standard of China. Interestingly, PCA further demonstrates that the dithizone-CTAB solution can be applied for not only effective identification and discrimination between Hg^2+^ and Cd^2+^, but their different concentrations (Fig. 3d). Furthermore, it’s worthy to note herein, the comparisons of the color responses of dithizone to Cd^2+^ and Hg^2+^ in the absence and presence of CTAB clearly show that the sensitivities of dithizone are significantly enhanced with the addition of CTAB (Fig. 3b,c). Therefore, the as-developed single-indicator-based sensing platform demonstrates not only great potentials in detection and differentiation of multiple metal ions, but semi-quantitative and even quantitative analysis of individuals with high sensitivity, based on the corresponding color response profiles, RGB recognition patterns, HCA/PCA, and EDs fitting curves.

**Figure 4:**
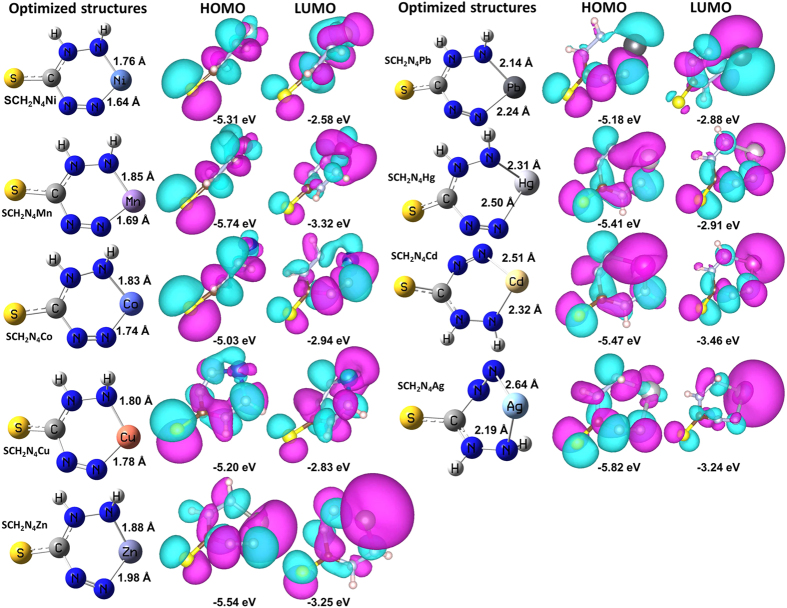
Density functional theory calculations. The optimized structures, HOMOs and LUMOs of the chelate structures of [SCH_2_N_4_]^2−^ and heavy metal ions, as well as the selected bond length parameters (Å).

To reveal the nature of distinct color responses of dithizone to diverse metal ions, of which lay the foundation for constructing the as-proposed triple-channel multidimensional sensing platform, we extend our efforts to apply the experimental means and density functional theory (DFT) calculations to study the interactions between the probe and heavy metal ions. Experimentally, the UV-vis absorption at the maximal wavelength (λ_max_) is mainly attributed to the electron transitions of HOMO to LUMO^43^,^44^,^45^,^46^. As shown in Fig. S6 (SI), discinct UV-vis spectra are observed with the addition of different metal ions, and the corresponding HOMO-LUMO energy gaps are calculated (see Table 1). But it’s hard to achieve useful differentiation information solely from these spectra. Nevertheless, the HOMO-LUMO energy gaps of the sensing system with each of these heavy metal ions could be calculated. Theoretically, DFT has been proven to be a powerful tool for determining the molecular structures and molecular orbitals^45^,^46^,^47^,^48^,^49^,^50^,^51^,^52^. Therefore, DFT calculations are applied to determine the chelate structures of the probe to heavy metal ions, and further for the corresponding HOMO to LUMO energy states. Based on the fact that dithizone decomposes to [SCH_2_N_4_]^2−^ in alkaline solution^29^, the chelates of [SCH_2_N_4_]^2−^ to nine heavy metal ions (expect Cr(VI)) are actually performed. Note that the special structure of Cr(VI) (i.e. Cr_2_O_7_^2−^) would produce completely different structures with the probe from the other nine metal ions, and therefore, Cr(VI) will not be discussed below. Through extensive DFT calculations using the optimized trial structures at B3LYP/6-31G* level, we obtained the accurate chelates of SCH_2_N_4_M (M = Ni, Mn, Co, Cu, Zn, Pb, Hg, Cd, and Ag) (Fig. 4). The shorter average distances of M-NH and M-N for Ni, Mn, Co, Cu, and Zn than that of Pb, Hg, Cd, and Ag reveal their stronger coordination capabilities with [SCH_2_N_4_]^2−^. The Cartesian coordinates of the optimized structures are summarized in Table S4 (SI). Moreover, for the purpose of clarifying the significant differences in the electronic distribution and transitions among the optimized chelates, their molecular orbitals have been theoretically investigated on the basis of DFT calculations with B3LYP/6-31G* level as well. As shown in Fig. 4, the corresponding frontier molecular orbital energies (i.e. HOMOs and LUMOs) and electronic distributions of the optimized chelates are found to be distinctively different. Their HOMO-LUMO gaps and the frontier molecular orbital energies are presented in Table 1 and Fig. S7 (SI). The calculated HOMO-LUMO gap increases in the order from 2.0 eV for SCH_2_N_4_Cd, 2.10 eV for SCH_2_N_4_Co, 2.29 eV for SCH_2_N_4_Zn, 2.30 eV for SCH_2_N_4_Pb, 2.37 eV for SCH_2_N_4_Cu, 2.42 eV for SCH_2_N_4_Mn, 2.50 eV for SCH_2_N_4_Hg, 2.58 eV for SCH_2_N_4_Ag, to 2.72 eV for SCH_2_N_4_Ni. Importantly, these calculated HOMO-LUMO gaps are found in high agreement with the experimental data (Table 1), which confirms the assumed chelating structures of the probe to metal ions. The combination of experimental characterizations and theoretical calculations demonstrate that the distinct color changes of the probe to heavy metal ions result from the different interactions, electron distributions and transitions.

The practical applicability of the as-developed sensing platform is preliminarily evaluated using real wastewater samples (taken from Yongjiang River in Ningbo, China). Firstly, the real water sample was spiked with the ten heavy metal ions at their standard concentrations of wastewater-discharge, and then subject to the same analysis process as that of in deionized water. As shown in Fig. S8 (SI), clearly distinct color response profiles of the sensor to each of the ten metal ions are observed, even by the naked eye. Similarly, the sextuple data are acquired to probe reproducibility of the sensing system and further for statistical analysis. Again, the as-developed triple-channel sensing platform exhibits distinct ΔRGB recognition patterns to the ten heavy metal ions and are well separated from each other based on PCA and HCA (Fig. S9 in SI). These findings demonstrate that the single-indicator-based triple-channel sensing platform can potentially be applied in analysis of real wastewater samples.

## Discussion

In summary, a novel concept to fabricate multidimensional sensing platforms is proposed through simply extracting more hiddern information, such as RGB alterations, from a single indicator that could show distinct color or fluorescence responses to diverse substances. As a proof-of-concept study, a triple-channel multi-ion analysis platform is developed solely using dithizone with assistance of a surfactant (i.e. CTAB) for hyperchromicity and sensitization. Through extracting the RGB alterations of dithizone and CTAB mixture to ten common heavy metal ions at their concentrations of wastewater-discharge of China, different recognition patterns (fingerprints) can be observed, and HCA/PCA further demonstrate its detection and discrimination capability. Moreover, this as-developed single-indicator-based sensing platform could as well as be applied in semiquantitative and even quantitative analysis of a specific metal ions with high sensitivity based on their corresponding color response profiles and EDs fitting curves, respectively. To reveal the nature of distinct color responses of dithizone to metal ions, DFT calculations are employed to determine their chelate structures and the corresponding HOMO-LUMO energy gaps. The high agreement of experimental characterizations and theoretical calculations demonstrate that the optimized chelates are credible, and the distinct color changes of the probe to heavy metal ions result from the different interactions, electron distributions and transitions. Compared to the traditional strategies for multidimensional sensing systems, the greatest advantage of the as-proposed approach is considered to be maximally simiplify the fabrication process. Notably, this work is only regarded as a preliminary step for exploration and application of the suggested single-indicator-based multidimensional sensing concept, and we are now focusing on extending its applications. The more recent research found that multiple proteins could also be detected and discriminated through applying a single Au nanoparticles-based probe, demonstrating great potentials of general applicability of the as-proposed concept, and the relevant results will be published separately elsewhere later.

## Methods

### Materials and instruments

Dithizone, cetyltrimethylammonium bromide (CTAB) and K_2_Cr_2_O_7_ were from Sinopharm Chemical Reagent Co., Ltd (Beijing, China). Mn(ClO_4_)_2_·6H_2_O, Ni(ClO_4_)_2_·6H_2_O, Cd(ClO_4_)_2_·6H_2_O, Hg(ClO_4_)_2_·3H_2_O and Zn(ClO_4_)_2_·6H_2_O were obtained from Strem Chemicals Inc. (Newburyport, USA). Pb(ClO_4_)_2_·3H_2_O and Co(ClO_4_)_2_·6H_2_O were purchased from Sigma-Aldrich Co. (USA). AgNO_3_ and 4-(2-hydroxyethyl)piperazine-1-ethanesulfonic acid (HEPES) were from Aladdin Reagent Co. Ltd (Shanghai, China). Cu(ClO_4_)_2_·6H_2_O was purchased from J&K Chemical Ltd. All chemicals were used as received without further purification. 96-well plates (Corning 3632) were obtained from Genetimes Technology. The stock solutions of metal ions were prepared using 50 mM HEPES (pH 7.4) buffer.

The pH measurements were performed using a PHS-3C pH meter. For all sensing experiments, imagings were acquired with a flatbed scanner (Epson Perfection V300) in 96-well plates. UV-vis absorption spectra were recorded using a Lambda 950 UV-vis spectrophotometer from Perkin Elmer. Fourier transform infra-red (FT-IR) spectroscopy was performed using a Nicolet 6700 spectrometer.

### Method for metal ions detection and discrimination

1.0 mL of dithizone solution (1.0 mM in 0.2 M NaOH) was firstly mixed with 24 mL of CTAB solution (0.125 mM in H_2_O), and then mixed with HEPES buffer that in the absence (control solutions) or in the presence of certain concentrations of metal ions (work solutions) at a volume ratio of 3:1. The final concentrations of dithizone, CTAB and HEPES are 30 μM, 90 μM and 10 mM, respectively.

300 μL of the control and work solutions were loaded into a 96-well plate, respectively, and the “before” (from the control solutions) and “after” (from the work solutions) images were acquired on an Epson Perfection V300 photo flatbed scanner. Difference maps were acquired by taking the difference of the RGB values from the center of the indicator solution (in 96-well plates) from the “before” and “after” images using the commercial Photoshop software.

The chemometric analysis was performed on the color difference vectors using the Multi-Variate Statistical Package (MVSP v.3.1, Kovach Computing); in all cases, hierarchical cluster analysis (HCA) and principal component analysis (PCA) were performed on the database library (Table S2) using the minimum variance for classification.

### Analysis of metal ions in real samples

Metal ions detection in wastewater from Yongjiang River (located in Ningbo, China) was taken as an example to preliminarily test the capability of the as-developed sensing platform for real samples. The analysis procedure was the same as the above description, just by using wastewater from Yongjiang River instead of deionized water.

### Calculations

The geometry optimizations of dithizone, dithizone products and the chelate structures of dithizone product and heavy metal ions, their molecular orbitals were performed by means of DFT methods using the Gaussian03 quantum chemistry package at the B3LYP/6-31G* level.

## Additional Information

**How to cite this article**: Leng, Y. *et al*. Single-indicator-based Multidimensional Sensing: Detection and Identification of Heavy Metal Ions and Understanding the Foundations from Experiment to Simulation. *Sci. Rep*. **6**, 25354; doi: 10.1038/srep25354 (2016).

## Supplementary Material

Supplementary Information

## Figures and Tables

**Table 1:** Comparison of the HOMO-LUMO energy gaps of the chelate structures of [SCH_2_N_4_]^2−^ and heavy metal ions from DFT calculations and UV-vis measurements.

	HOMO-LUMO energy gaps (eV)	
	DFT calculations	Experimental (λ_max_/nm)
SCH_2_N_4_Cd	2.01	2.17 (570 nm)
SCH_2_N_4_Co	2.09	2.12 (585 nm)
SCH_2_N_4_Zn	2.29	2.29 (541 nm)
SCH_2_N_4_Pb	2.30	2.38 (520 nm)
SCH_2_N_4_Cu	2.37	2.30 (540 nm)
SCH_2_N_4_Mn	2.42	2.52 (492 nm)
SCH_2_N_4_Hg	2.50	2.55 (485 nm)
SCH_2_N_4_Ag	2.58	2.55 (487 nm)
SCH_2_N_4_Ni	2.73	2.73 (455 nm)

Note: experimental HOMO-LUMO energy gaps = hc/λ_max_, h = 6.626 × 10^−34^ J.s, c = 3 × 10^8^ m/s.
