# Supplementary material for: Single-indicator-based Multidimensional Sensing: Detection and Identification of Heavy Metal Ions and Understanding the Foundations from Experiment to Simulation
Source: Sci Rep. 2016 May 5;6:25354. doi: 10.1038/srep25354 (PMC4857083; doi:10.1038/srep25354)
Supplement: Supplementary Information [file srep25354-s1.pdf]

Supplementary Information

Single-indicator-based Multidimensional Sensing: Detection and Identification of Heavy Metal Ions and Understanding the Foundations from Experiment to Simulation

Yumin Leng[1,2], Sihua Qian[1], Yuhui Wang[1,]*, Cheng Lu[2], Xiaoxu Ji[2], Zhiwen Lu[2] & Hengwei Lin[1,]*

[1]Ningbo Institute of Materials Technology & Engineering (NIMTE), Chinese Academy of Sciences, Ningbo 315201, China

[2]College of Physics and Electronic Engineering, Nanyang Normal University, Nanyang 473061, China

*E-mail: linhengwei@nimte.ac.cn

Supporting Figures

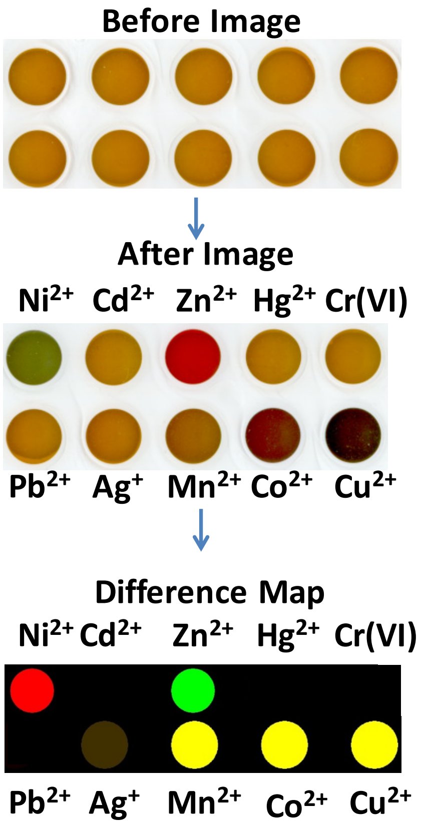

Figure S1. Color images of the dithizone solution before and after exposure to ten heavy metal ions at standard concentrations of wastewater-discharge of China (see Table S1). For purposes of visualization, the color range of the map was expanded from 4 to 8 bits per color (RGB range of 4−19 expanded to 0−255).

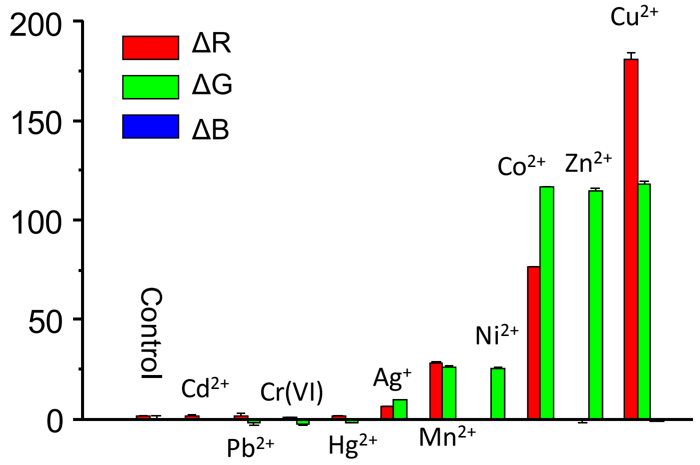

Figure S2. The RGB alterations (fingerprints) of the dithizone solution to ten heavy metal ions at their wastewater-discharge standard concentrations of China and a control. All experiments were run in triplicate. Control, Cd^{2+}, Pb^{2+}, Cr(VI) and Hg^{2+} cannot be differentiated.

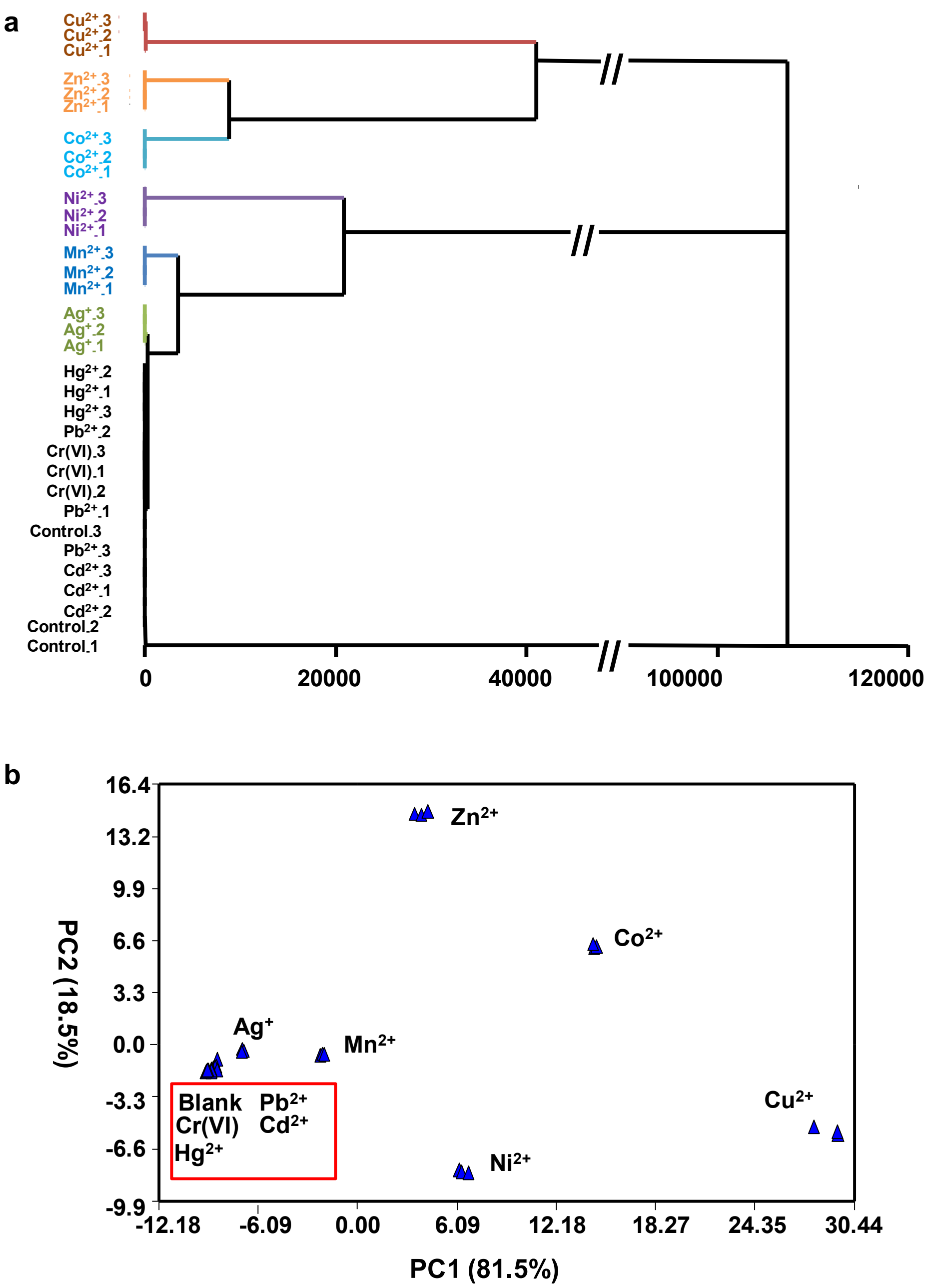

Figure S3. (a) HCA, and (b) PCA for the dithizone solution analysis of ten heavy metal ions at wastewater-discharge standard concentrations of China and a control based on the ΔRGB values obtained from the "before" and "after" images. All experiments were run in triplicate. Confusions of Control, Cd^{2+}, Pb^{2+}, Cr(VI) and Hg^{2+} in classification were observed.

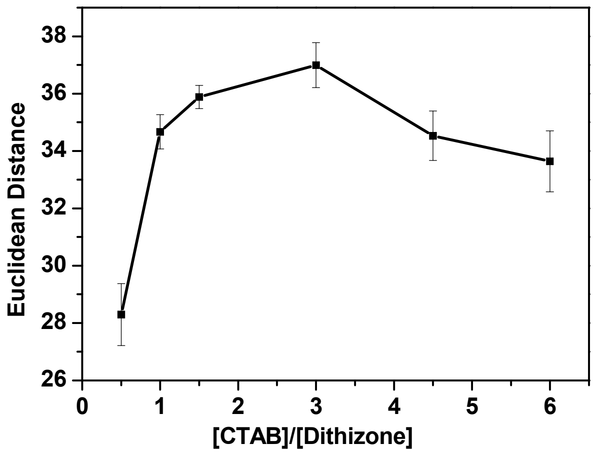

Figure S4. The total Euclidean distances of the dithizone solution mixed with CTAB at different ratios response to 5 μM Cd^{2+}. The ratios of CTAB to Dithizone are 1:2, 1:1, 3:2, 3:1, 4.5:1 and 6:1. The experiments were performed in triplicate with 30 μM dithizone and 5 μM Cd^{2+} under physiological conditions (10 mM HEPES buffer at pH 7.4). The error bars represent the standard deviation of triplicate experiments.

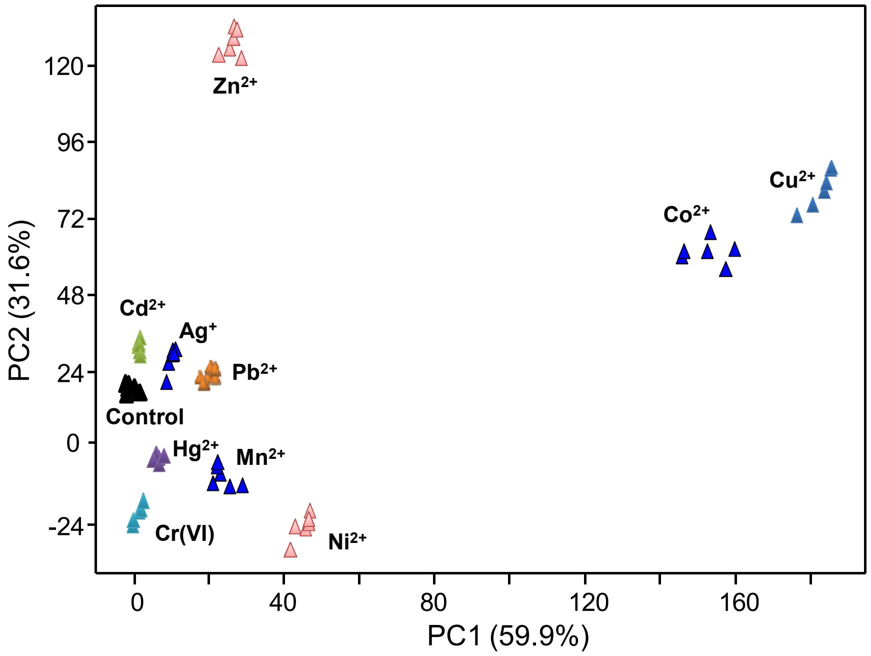

Figure S5. PCA score plot for the analysis of ten heavy metal ions using the as-developed single-indicator-based multidimensional sensing platform at their standard concentrations of wastewater-discharge of China.

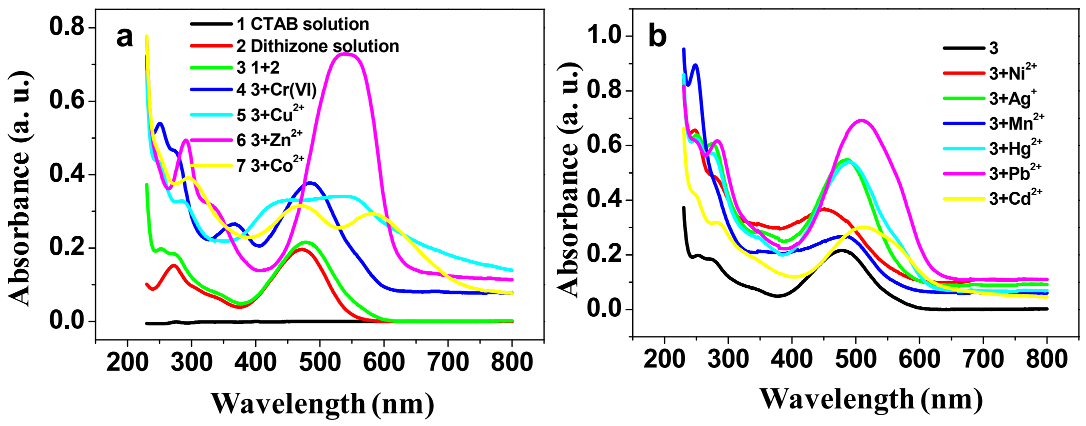

Figure S6. UV-vis absorption spectra of the CTAB solution, dithizone solution, their mixture, and the mixture in the presence of each of ten heavy metal ions at their wastewater-discharge standard concentrations of China.

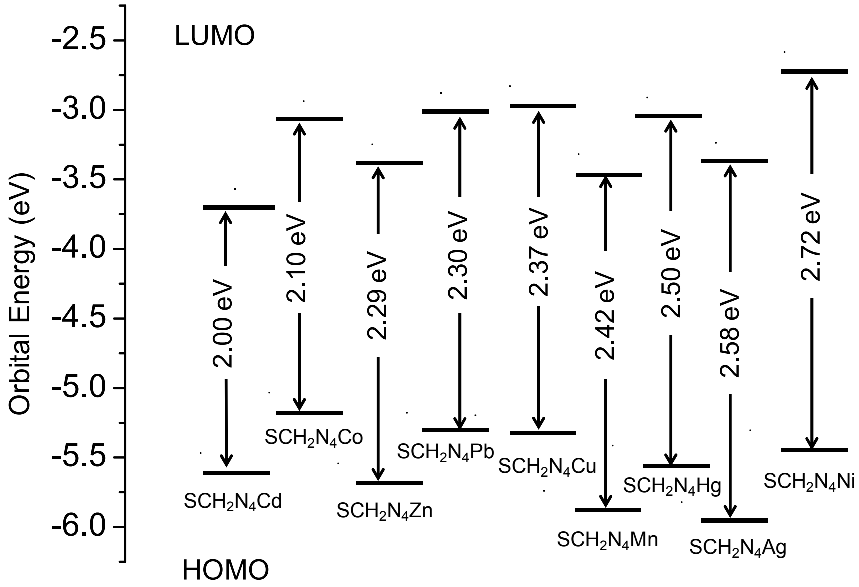

Figure S7. The frontier molecular orbital energies of the chelate structures of $[SCH_2N_4]^{2-}$ and heavy metal ions, as well as the gaps between HOMO and LUMO.

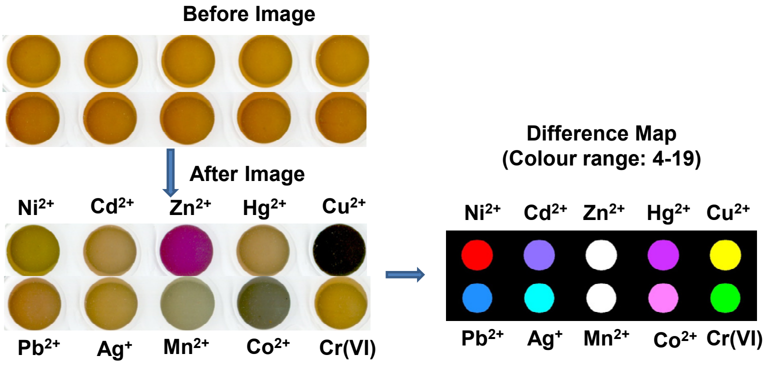

Figure S8. Color images of the sensor before and after exposure to ten heavy metal ions at their standard concentrations of wastewater-discharge in wastewater from Yongjiang River. For purposes of visualization, the color range of the map was expanded from 4 to 8 bits per color (RGB range of 4−19 expanded to 0−255).

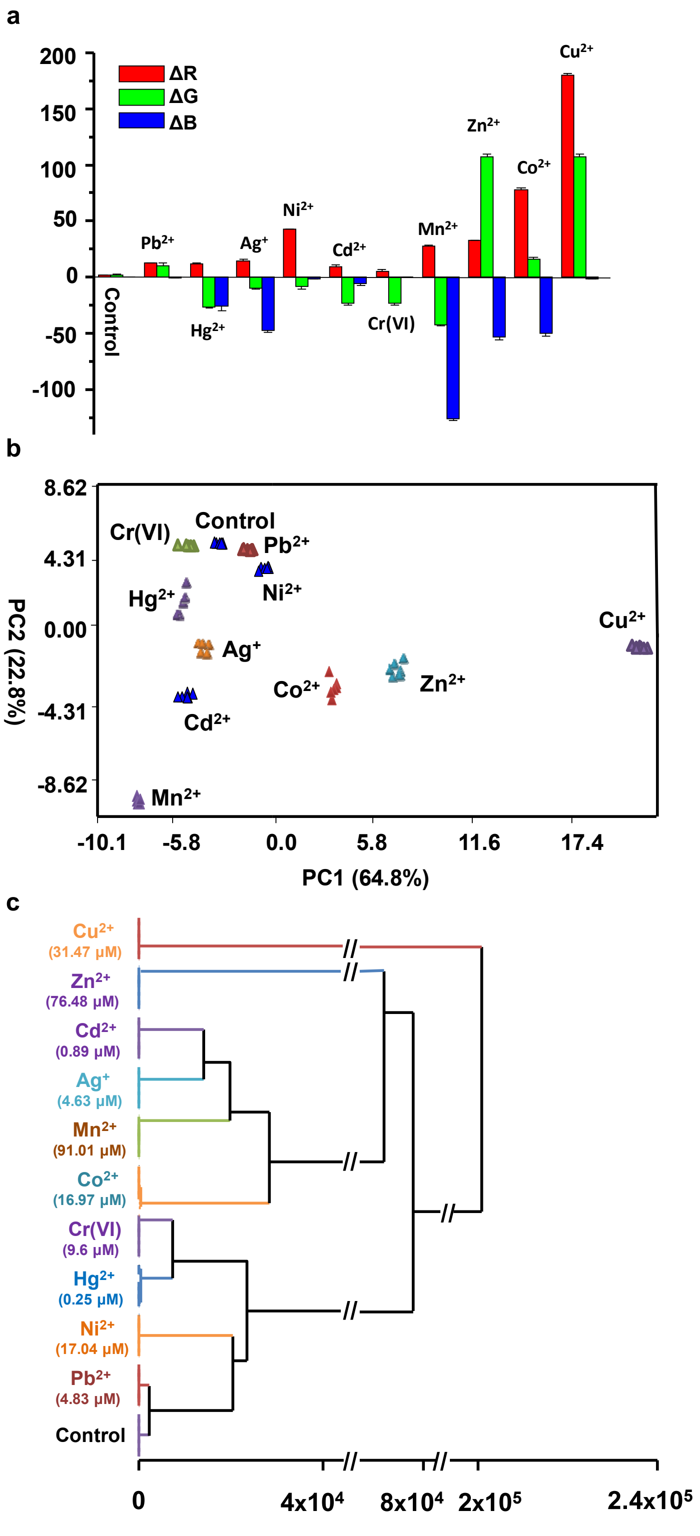

Figure S9. (a) Recognition patterns, (b) PCA score plot, and (c) HCA for the analysis of the ten heavy metal ions at their standard concentrations of wastewater-discharge of China in wastewater from Yongjiang River. Error bars in Figure a represent standard deviations of six parallel measurements.

Supporting Tables

Table S1. Wastewater-discharge standard concentrations of China for ten heavy metal ions (referred to Chinese National Standards: Integrated Wastewater-Discharge Standard (GB8978-1996)).

**Heavy Metal Ions**	**Wastewater Discharge Standard**	
	**mg/L**	**μM**
Hg^{2+}	0.05	0.25
Cd^{2+}	0.1	0.89
Pb^{2+}	1.0	4.83
Cr(VI)	0.5	9.61
Co^{2+}	1.0	16.97
Ni^{2+}	1.0	17.04
Cu^{2+}	2.0	31.47
Zn^{2+}	5.0	76.48
Mn^{2+}	5.0	91.01
Ag^{+}	0.5	4.63

Table S2. Database of the RGB alterations of the dithizone and CTAB mixture to ten heavy metal ions at their standard concentrations of wastewater-discharge of China and a control.

	R	G	B		R	G	B
Control-1	-1.7827	4.0975	0.0000	Hg^{2+}-1	6.7532	-17.9869	-12.6545
Control-2	-1.4054	5.3579	0.0100	Hg^{2+}-2	5.9866	-16.4622	-11.6322
Control-3	-2.3152	5.4956	0.0000	Hg^{2+}-3	5.8962	-16.6566	-9.9973
Control-4	-1.9621	2.1171	-0.1477	Hg^{2+}-4	5.5632	-18.6361	-12.0458
Control-5	0.0791	4.3592	0.0008	Hg^{2+}-5	6.6063	-19.7450	-10.8098
Control-6	1.4959	2.5828	-0.1997	Hg^{2+}-6	7.9604	-17.0518	-13.6988
Co^{2+}-1	157.2967	41.3338	-1.7290	Mn^{2+}-1	28.8890	-26.5408	-93.7995
Co^{2+}-2	145.7337	45.3188	-1.6677	Mn^{2+}-2	25.4102	-26.6594	-96.0769
Co^{2+}-3	159.6144	47.6667	-1.6355	Mn^{2+}-3	21.0583	-25.7631	-97.8830
Co^{2+}-4	152.4157	47.1482	-1.4773	Mn^{2+}-4	22.8387	-22.8676	-106.3502
Co^{2+}-5	153.2831	53.0550	-1.4647	Mn^{2+}-5	22.0374	-20.2964	-97.6930
Co^{2+}-6	146.2841	47.1434	-1.0668	Mn^{2+}-6	22.2165	-19.1120	-102.3072
Ni^{2+}-1	41.6290	-46.7310	-0.0005	Zn^{2+}-1	22.5654	108.6071	-115.4632
Ni^{2+}-2	42.7591	-39.3512	-0.0681	Zn^{2+}-2	25.5225	110.4680	-117.3107
Ni^{2+}-3	45.7272	-40.1603	0.0000	Zn^{2+}-3	26.4943	113.7882	-111.7714
Ni^{2+}-4	46.4878	-38.2271	0.0000	Zn^{2+}-4	26.6338	117.4619	-113.8335
Ni^{2+}-5	46.8490	-34.3670	0.0000	Zn^{2+}-5	27.4391	116.6021	-115.4069
Ni^{2+}-6	46.5948	-37.0334	-0.0048	Zn^{2+}-6	28.5800	107.5391	-111.8367
Cd^{2+}-1	1.0766	17.1630	-0.0080	Pb^{2+}-1	19.4651	7.9875	-43.4527
Cd^{2+}-2	1.5177	15.5232	0.0000	Pb^{2+}-2	17.3851	7.9657	-36.9847
Cd^{2+}-3	1.2534	18.2364	-0.0048	Pb^{2+}-3	20.2040	10.9638	-39.6546
Cd^{2+}-4	1.7565	20.0220	0.0000	Pb^{2+}-4	21.4657	7.6775	-38.5347
Cd^{2+}-5	1.4531	17.6355	-0.0050	Pb^{2+}-5	21.3656	10.5632	-40.4516
Cd^{2+}-6	1.6530	14.2347	0.0000	Pb^{2+}-6	18.3426	5.8910	-42.1472
Cr(VI)-1	-0.2253	-38.9779	0.0018	Ag^{+}-1	8.6230	5.9141	-38.2120
Cr(VI)-2	-0.1226	-37.4544	0.0000	Ag^{+}-2	9.2894	11.9425	-42.5157
Cr(VI)-3	1.2929	-34.8749	-0.0020	Ag^{+}-3	10.2321	14.7259	-47.1982
Cr(VI)-4	1.9694	-34.1367	0.0000	Ag^{+}-4	11.0598	16.1784	-46.7538
Cr(VI)-5	2.5614	-31.3888	-0.0038	Ag^{+}-5	10.2585	16.0392	-46.1178
Cr(VI)-6	2.5549	-31.2542	0.0000	Ag^{+}-6	10.5870	15.0543	-48.7737
Cu^{2+}-1	176.3017	58.1899	-0.1625				
Cu^{2+}-2	180.4758	61.6056	-0.0819				
Cu^{2+}-3	183.5685	65.7830	-0.1756				
Cu^{2+}-4	184.0980	68.4635	-0.0668				
Cu^{2+}-5	185.1226	72.9754	-0.1861				
Cu^{2+}-6	185.4469	73.3373	0.0000				

Table S3. The equation and parameters for limit of detection (LOD) calculation.

$Y = A + S \times X$						
Parameter	A	S	R	SD	N	P
Hg^{2+}	18.21118	12.71978	0.99946	0.56191	6	<0.0001
Cd^{2+}	9.03225	9.92698	0.99985	0.46991	6	<0.0001

A = Y-intercept, S = slope, R = correlation, SD = standard deviation, N = number of data points, P = probability value.

The limit of detection (LOD) could be obtained by the above equation and parameters.

$LOD = 3 \times SD/S$

$LOD_{Hg^{2+}} = 3 \times 0.56191/12.71978 = 0.13\ \mu M$

$LOD_{Cd^{2+}} = 3 \times 0.46991/9.92698 = 0.14\ \mu M$

Table S4. The Cartesian coordinates of the chelate structures of $[SCH_2N_4]^{2-}$ and different heavy metal ions.

Conformer Å		X	Y	Z	Conformer Å		X	Y	Z
SCH_2N_4Ni	N	0.819437	1.352584	-0.066088	SCH_2N_4Pb	N	-0.200790	1.428472	0.481742
	H	1.053446	2.156162	0.520918		H	-0.048247	2.230589	1.088389
	N	-0.545011	1.132531	0.099376		N	-1.574376	1.225206	0.341824
	H	-1.133990	1.953322	-0.000124		H	-2.108798	2.013206	-0.018268
	C	-1.251459	-0.029665	0.058224		C	-2.210777	0.041724	0.211127
	S	-2.919086	-0.060615	-0.063681		S	-3.698080	-0.144948	-0.504844
	N	-0.513062	-1.253435	0.104590		N	-1.566211	-1.084980	0.826553
	N	0.703660	-1.284758	0.059224		N	-0.410875	-1.404020	0.557582
	Ni	1.822840	-0.092504	-0.043963		Pb	1.229960	-0.040582	-0.118455
SCH_2N_4Mn	N	0.833004	1.339535	0.000728	SCH_2N_4Hg	N	-0.216010	1.432585	0.879520
	H	1.020084	2.285916	0.325307		H	0.162655	2.389670	1.226128
	N	-0.535510	1.126256	0.059738		N	-1.478792	1.273368	0.359615
	H	-1.132976	1.939181	-0.037443		H	-1.954591	2.058060	-0.087305
	C	-1.214712	-0.060326	0.033601		C	-2.164375	0.129333	0.238597
	S	-2.892447	-0.066905	-0.051123		S	-3.454750	-0.177494	-0.743510
	N	-0.487904	-1.260202	0.092649		N	-1.628408	-1.024809	1.071788
	N	0.756085	-1.248592	0.055724		N	-0.514629	-1.415861	1.021691
	Mn	1.988824	-0.099666	-0.045335		Hg	1.215555	-0.053010	-0.175032
SCH_2N_4Co	N	0.809927	1.385462	0.060691	SCH_2N_4Cd	N	0.214025	1.397214	0.845019
	H	1.061848	2.050550	0.778090		H	0.288473	2.346655	1.208358
	N	-0.554999	1.156140	0.165110		N	-1.066297	1.260850	0.362892
	H	-1.135549	1.955132	-0.027861		H	-1.547822	2.058929	-0.053123
	C	-1.231356	0.009195	0.111073		C	-1.767392	0.126282	0.241621
	S	-2.860728	-0.073772	-0.160979		S	-3.091584	-0.148208	-0.704380
	N	-0.526676	-1.242616	0.300603		N	-1.217305	-1.049420	1.034490
	N	0.665000	-1.325299	0.151491		N	-0.109866	-1.450400	0.941054
	Co	1.869656	-0.099863	-0.132825		Cd	1.595524	-0.081243	-0.283731
SCH_2N_4Cu	N	0.815616	1.434402	0.080800	SCH_2N_4Ag	N	-0.334392	-1.297078	0.879106
	H	1.060682	2.176157	0.735558		H	-0.327843	-0.875842	1.811862
	N	-0.546003	1.172078	0.271012		N	0.978242	-1.184351	0.392492

	H	-1.159798	1.978950	0.174965		H	1.313955	-2.023446	-0.073392
	C	-1.226698	0.006291	0.139712		C	1.755155	-0.107532	0.206339
	S	-2.852799	-0.061902	-0.228030		S	3.196120	-0.026921	-0.593123
	N	-0.536684	-1.232652	0.432989		N	1.231460	1.120872	0.857140
	N	0.632936	-1.442743	0.192097		N	0.250300	1.696383	0.525806
	Cu	1.742863	-0.093794	-0.170297		Ag	-1.649664	0.034563	-0.256773
SCH_2N_4Zn	N	0.819522	1.458100	0.319480					
	H	1.119801	2.092937	1.054143					
	N	-0.568472	1.271670	0.409798					
	H	-1.157216	2.061132	0.138459					
	C	-1.185740	0.081094	0.247198					
	S	-2.699260	-0.101910	-0.409191					
	N	-0.552568	-1.124970	0.759496					
	N	0.557205	-1.563369	0.484345					
	Zn	1.618340	-0.110003	-0.331352					
